# The Field of Science

**Published:** 1894-01-13

**Authors:** 


### The Field of Science.

The necessity for the establishment of a pathological laboratory and museum in connection with the London lunatic asylums has long been felt, and decisive steps have now been taken to establish the same. The institution will be situated at Claybury; and the pathologist, when appointed to this post, will be supplied with material from the local and other asylums. The decision for this much-needed addition for the furtherance of cerebral pathology was made at the instigation of the London County Council. In future, clinical and experimental investigators on this subject will have greater facilities offered to them, and ready to hand for work, instead of being hampered, as in the past, for want of any systematic supply of material.

A curious pathological specimen illustrated one of M. Lancereaux's recent lectures at the Academy of Medicine. This exhibition was no other than the lungs of a man, whose trade—that of a polisher of electric light carbons, had transformed these particular organs themselves into veritable blocks of carbon. Previously to this polishing business the deceased had been a vigorous stonemason from boyhood up to his thirty-sixth year. Later on in life he served in a workshop, eight metres long by seven broad, where, with some others he was engaged in polishing carbons for electric light purposes. The little room was so sparsely ventilated that there was no outlet for the charcoal and the stone dust with which the air was impregnated. Sometimes the workers could not distinguish one another through the density of the atmosphere in which they lived through the daytime. It is hardly to be wondered at, therefore, under these circumstances that the lungs of a man working in such conditions should become a receptacle for the pernicious surroundings. And yet surely the result in so tragic and fatal a form might have been averted had a few simple precautionary measures been brought to bear upon the field of operations—the use of a respirator, for instance, more ventilation, and a greater air space for the men to breathe in who are employed in this particular trade.

Russian conservative instincts are fast giving way in matters medical. Up to the present time the old Nuremberg system of weights and measures has been steadily adhered to throughout the Czar's domains. In all medical and pharmaceutical measure in future the metric system, it is announced, is to be made compulsory by a recent regulation of Russian law.

With a view to eliminating quackery in America and establishing soundness in medical practice, a society has recently been started across the Atlantic entitled "The American Electro-Therapeutic Association." The functions of this body are directed towards the investigations of such work as has already been accomplised in the application of electricity to the cure of human disease, and for the furtherance of any real work so far done on these lines. Mr. Newman Lawrence, whose writings have aroused an interest on this subject in Europe as in America, is anxious to found a representative institution in England based on the same principles.

The much-vexed question which the Worthing sewage has presented for so many months now seems at length as if it might mee twith a successful solution. The method of purifying sewage matter by electricity to which we have made lengthy reference in these pages. as being the invention of the French scientist, M. Hermite, was brought before the Worthing Town Council last month. Previously to this a representative deputation had waited upon M. Hermite himself in London, among whom was Councillor Dr. John Goldsmith, at whose instigation this plan, in the first instance, was put into execution. After going into the drainage question in its various and complicated aspects, M. Hermite has consented to conduct experiments at Worthing for one month. The Town Council have granted the sum of £200, required to carry out the same. The French scientist will start operations forthwith, one side of West Street having been chosen for the experimental ground. Sir H. Roscoe and Dr. Ruffer have consented to test the value of the experiments, chemically and bacteriologically.

